# Tuberculosis at a university hospital, Thailand: A surprising incidence of TB among a new generation of highly exposed health care workers who may be asymptomatic

**DOI:** 10.1371/journal.pone.0273027

**Published:** 2022-08-24

**Authors:** Pichanon Mingchay, Leilani Paitoonpong, Kamon Kawkitinarong, Pirapon June Ohata, Gompol Suwanpimolkul

**Affiliations:** 1 Faculty of Medicine, Department of Medicine, Chulalongkorn University and King Chulalongkorn Memorial Hospital, Thai Red Cross Society, Bangkok, Thailand; 2 Emerging Infectious Diseases Clinical Center, Thai Red Cross Society, Bangkok, Thailand; 3 Tuberculosis Research Unit, Chulalongkorn University, Bangkok, Thailand; 4 HIV-NAT, Thai Red Cross–AIDS Research Centre, Bangkok, Thailand; The University of Georgia, UNITED STATES

## Abstract

**Objectives:**

There are a few reports of TB disease among healthcare workers (HCWs) in Thailand. The authors assessed the demographic data and incidence of overall TB disease including specific profession among HCWs in order to reduce the incidence rate.

**Methods:**

This was a descriptive, cross-sectional study of 195 HCWs at King Chulalongkorn Memorial Hospital (KCMH), Bangkok, Thailand, who had TB disease from 2003–2020.

**Results:**

The average incidence of TB disease in HCWs at KCMH was 164 per 100,000 HCWs with 95% confidential interval [CI], 107 to 220; (94.4% had pulmonary TB). Most of the HCWs were from a young age group (43.08% were 20–29 years old) and the duration of work was short (58.82% had worked at KCMH for less than 5 years). Radiological technicians had the highest incidence of TB, followed by supportive teams, scientists, and physicians (429, 241, 205, and 193 per 100,000 HCWs according to their profession, respectively). Seventy five percent of TB disease was found in physicians who worked at KCMH within 2 years. TB incidence was highest among residents (688 per 100,000 residents) in a subgroup of physicians.

**Conclusions:**

The incidence of TB disease in HCWs was close to the general population of Thailand (153 per 100,000 population with 95% CI, 116 to 195). A higher incidence was observed in the profession that had contact with TB patients and their specimens. A high proportion of asymptomatic HCWs also had TB disease and TB incidence was found in a new generation of HCWs who have worked for less than 5 years. More rigorous epidemiology investigations are needed to establish a definitive relation to subsequently developing TB after working in a hospital. The authors suggest active surveillance in all new incoming HCWs and TB preventive therapy should also be provided to recent converters.

## Introduction

Tuberculosis (TB) is a communicable disease that is a major cause of illness, 1 of the top 10 causes of deaths worldwide and the leading cause of death from a single infectious agent (ranking above HIV/AIDS). In 2014, the End TB Strategy was a political declaration agreed by all United Nation member states (UN) to reduce the number of TB cases and deaths globally. This Declaration aimed to reduce the number of TB deaths by 95% and TB incidence by 90% by 2035 [1].

In the general population, many factors related to increased TB infection were bacillary load, close contact to index case, recent TB immunosuppressive condition (e.g., human immunodeficiency virus (HIV), immune mediated inflammatory disorder, treatment with immunosuppressive agents), malnutrition, diabetes, chronic renal failure, transplantation, silicosis, tobacco and alcohol use [2–5]. Health care workers (HCWs) were also reported to be one of the risk factors that was associated with TB incidence [5].

A systematic review showed the prevalence of latent TB infection (LTBI) among HCWs in low- and middle-income countries was 54% (range 33% to 79%). The annual risk of LTBI ranged from 0.5% to 14.3%, and the incidence of TB disease in HCWs ranged from 69 to 5,780 per 100,000. The attributable risk for TB in HCWs, compared to the risk in the general population, ranged from 25 to 5,361 per 100,000 per year [6]. Five years later, another systematic review which analyzed LTBI and TB among HCWs in a worldwide setting (both high- and low-income countries) reported HCWs were at higher-than-average risk for TB compared to the general population (RR 2.97, 95%CI 2.43–2.51) [7]. Thus, TB in HCWs continue to be a major concern in health care facilities.

TB is endemic in Thailand. According to the data in 2019, Thailand is 1 of the 14 countries that has a high TB burden (total TB incidence was 153 per 100,000 population with 95% confidential interval [CI], 116 to 195), high incidence of MDR TB (5.7 per 100,000 population with 95% confidential interval [CI], 3.3 to 8.8), and TB in HIV-infected population (15 per 100,000 population with 95% confidential interval [CI], 12 to 20) [1]. There are a few reports on TB among HCWs in Thailand [8–10]. All reports showed that there was a higher incidence of TB in HCWs compared to the general population [8–10]. Ramathibodi Hospital and Chiang Mai Hospital reported a high proportion of HCWs who have worked less than 5 years had TB disease (50.5% and 30.3%, respectively) [8,10]. Most of them were middle aged (40.4% was 30–39 years old and 28.4% was 20–29 years old) [8]. In addition, 22.9% of TB disease in HCWs were asymptomatic but were detected to have TB disease at their annual health checkup [8].

King Chulalongkorn Memorial Hospital (KCMH) is one of the leading university hospitals and referral centers in Thailand with 1,500 beds capacity (1,500,000 of outpatients and 50,000 of inpatients per year). A previous report of TB in HCWs at KCMH was published in 2005 which reported the TB incidence rate to be 188 per 100,000 person-years [9].

This study assessed the incidence of TB in HCWs in overall setting and within specific professions. Demographic data, previously known risk factors (e.g., age, sex, body mass index (BMI), underlying disease, previous history of TB, department of work, and duration of work), and characteristics of TB (e.g., classification, clinical presentation, results of investigation, and drug resistant pattern) were also explored.

## Methods

This was a single center, descriptive, cross-sectional study that was conducted at KCMH, Bangkok, Thailand. The study proposal was submitted and approved by the ethical committee at the Faculty of Medicine, Chulalongkorn University (IRB No.7/2563). The study was conducted in accordance with Good Clinical Practice guidelines and the principles of the Declaration of Helsinki. Prior to the screening process, all eligible candidates > 18 years old received information pertaining to the study and provided written informed consent form. All HCWs who voluntarily consented and diagnosed with TB disease during 2003–2020 at KCMH were analyzed. Personnel who had active TB treatment with any regimen during the period when they started to work at KCMH, under the age of 18 years, lost to follow-up during treatment, or death due to other cause which was not related to TB were excluded from the study. On the other hand, personnel who had completed their treatment course and was cured of TB before joining KCMH were included in the study. The hospital has a screening program for new staff before they started to work such as screening using a questionnaire and chest radiography. If TB was detected at the screening process, they would be diagnosed with TB prior to working and were excluded from the study. Tuberculin skin Test (TST) and Interferon-gamma release assays (IGRA) were not performed in all HCWs who joined the hospital in early 2000 due to limited resources and interpretation of the results. Thus, immunologic markers were not included in the inclusion criteria. Thailand’s EPI (Expanded Program on Immunization) provided Bacille Calmette-Guerin (BCG) vaccines to all Thai citizens at birth. HCWs were defined as people involved in actions whose primary intention were to promote health (e.g., physicians, nurses, and pharmacists) and other personnel working in the hospital who may be in contact with TB patients or infectious materials from TB patients (e.g., scientists, medical technologists, housekeepers, transporters, and office workers). TB disease was defined as having confirmed evidence of culture and/or molecular outcome (e.g., PCR for TB) for TB (both intrapulmonary TB and extrapulmonary TB) or expert opinion who decided to treat for TB using clinical judgment in a setting that had negative culture and/or molecular result. Asymptomatic HCWs who had abnormal chest radiography at their annual checkup and positive microbiological evidence for TB (culture or molecular study), or from expert judgment were classified as having TB disease. The authors reviewed the registered database (both electronic and paper–based medical records) at the airborne infection isolation clinic and data from the infection control committee for TB disease in HCWs. The data from the human resource department of KCMH from 2003–2020 for all HCWs, including HCWs in a specific profession, were gathered. The data collected were categorized into 3 groups: i) personal information (e.g., sex, age, BMI, and underlying disease), ii) Occupational information (e.g., profession, department of work, and duration of work), and iii) Tuberculosis information (e.g., type of TB, previous history of TB, clinical presentation, radiological finding, microbiological evidence, drug resistant profile, and treatment result). Statistical analyses were done using SPSS Statistics for Windows, Version 17.0. Chicago: SPSS Inc. (Chulalongkorn University, Thailand). The demographic data was shown as categorial variables and presented as count, proportion, and percentage (including sex, age, BMI, underlying disease, department of work, duration of work, type of TB, previous history of TB, clinical presentation, radiological finding, microbiological evidence, drug resistant profile, and treatment result). The authors calculated the incidence of overall TB in HCWs and specific profession by number of annual TB disease cases or annual TB disease cases in specific profession divided by the number of total HCWs or specific profession in the same year. Average incidence rate was calculated by average incidence per year to present the overview of TB disease among HCW at KCMH and by specific profession. In addition, 95% confidential interval [CI] was calculated by average incidence rate and standard deviation.

## Results

From January 2003 to December 2020, 195 HCWs were diagnosed with TB at KCMH and recruited into the study. No one met the exclusion criteria. The information of TB disease in workers was retrieved from the registered database (both electronic and paper–based medical records) at the airborne infection isolation clinic, and data from the infection control committee. Unfortunately, some of the demographic data were lost prior to 2012 because that was when the paper-based medical records were changed to electronic medical record in 2018. As a result of this, some of the paper-based medical records were destroyed if the patient did not have any hospital visit for more than 5 years. The age of the HCWs and department of work were available in 2003 at time of recruitment but the sex data were available from 2005. Other demographic data (e.g., BMI, underlying disease, duration of work, type of TB, previous history of TB, clinical presentation, radiological finding, microbiological evidence, drug resistant profile, and treatment result) were available from 2012. For incidence calculation, the data of overall HCWs and HCWs in specific profession at KCMH were received from the human resource department at KCMH since 2013. Therefore, the incidence rate was calculated from 2013 to 2020.

**Fig 1:**
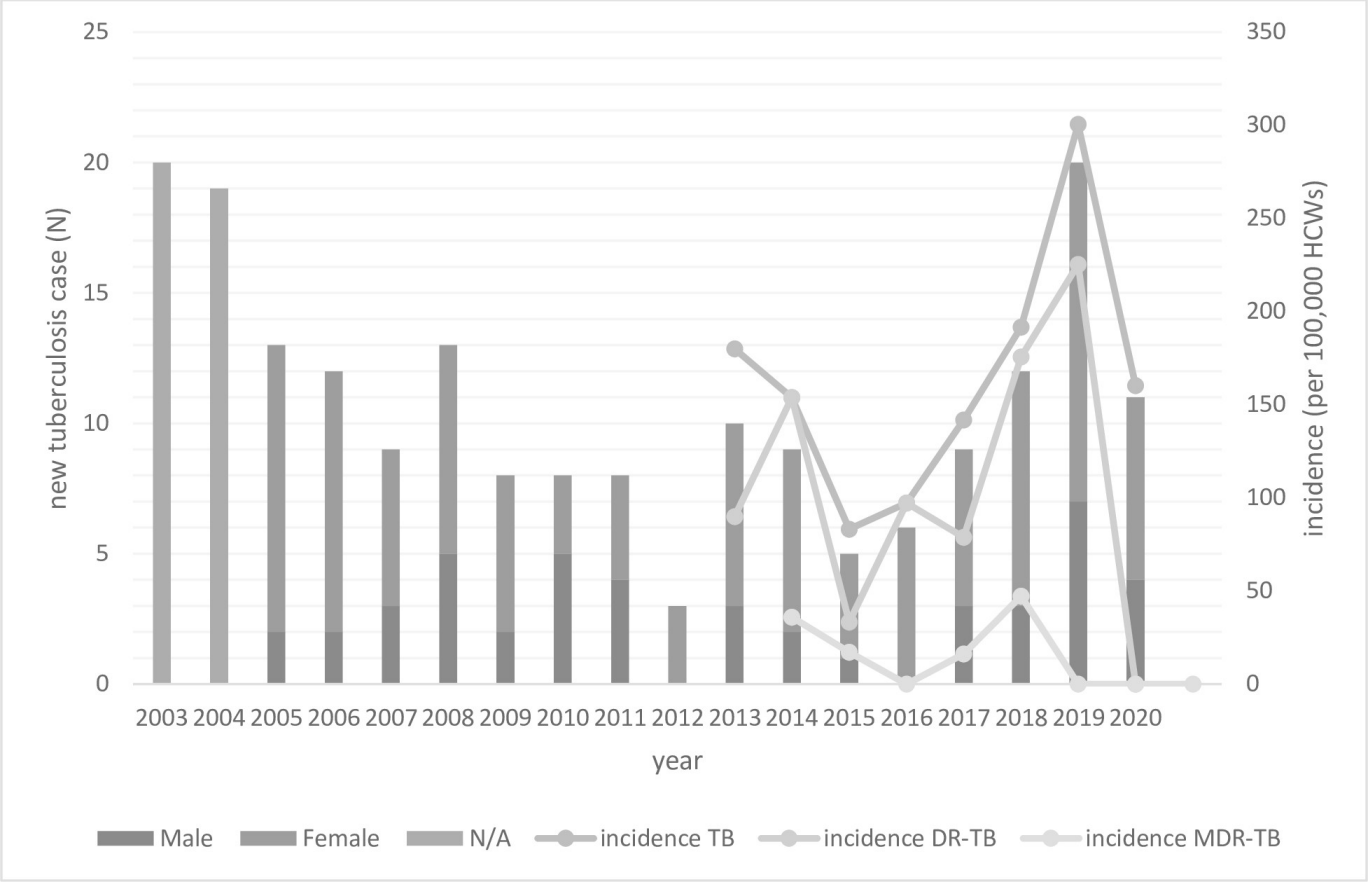
New tuberculosis cases, incidence of overall TB disease, DR-TB, and MDR-TB in HCWs at KCMH from 2013–2020. TB: Tuberculosis, DR-TB: Ddrug- resistant tuberculosis, MDR-TB: Multi-drug resistant tuberculosis, HCWs: Healthcare workers, KCMH: King Chulalongkorn Memorial Hospital, N/A: Not available. * incidence per 100,000 HCWs was calculated from 2013–2020.

The incidence of TB in HCWs had increased since 2013. From 2003 to 2020, the number of TB disease in HCWs at KCMH were 20, 19, 13, 12, 9,13, 8, 8, 8, 3, 10, 9, 5, 6, 9, 12, 20, 11, respectively. Total HCWs at risk was collected since 2013 (5,559, 5,851, 6,011, 6,168, 6,350, 6,263, 6,657, 6,866 HCWs, respectively). Therefore, the overall incidence of TB in HCWs was calculated from 2013 to 2020 (180, 154, 83, 97, 142, 192, 300, 160 per 100,000 HCWs respectively). The average incidence of TB disease in HCWs was 164 per 100,000 HCWs (95% confidential interval [CI], 107 to 220). In addition, the incidence of drug resisitant TB disease (DR-TB) from 2013 to 2020 was 90, 154, 33, 97, 79, 176, 225, and 0 per 100,000 HCWs, respectively (average 107 per 100,000 HCWs; 95% CI, 53 to 160). In contrast, the incidence of MDR-TB disease was 36, 17, 0, 16, 47, 0, 0, 0 per 100,000 HCWs, respectively (average 15 per 100,000 HCWs; 95% CI, 3 to 27). The incidence of MDR-TB in the last 3 years was 0. As previously mentioned, the incidence was calculated from 2013 to 2020 because there was insufficient data prior to 2013. There was a higher adjusted proportion of TB disease in males compared to females (the male to female ratio of overall HCWs was 2:8). Data from 2003–2004 only showed the number of TB disease cases; the sex of the HCWs were not recorded (Fig 1).

Out of 195 HCWs by proportion, 184 (94.4%) had pulmonary TB and only 11 HCWs (5.6%) had extrapulmonary TB. Eight cases (4.1%) had TB in the lymph node and 3 cases (1.5%) had TB in the pleura. Fifteen HCWs (8.15%) had a combination of both pulmonary and extrapulmonary TB. Nine cases (4.6%) had pulmonary TB and TB in the pleura, 5 cases (2.6%) had pulmonary TB and TB in the lymph node, and 1 case (0.5%) had pulmonary TB and TB in the nasopharynx.

Most of the HCWs that were diagnosed with TB disease were from the young age groups. Eighty-four of diagnosed HCWs (43.08%) were 20–29 years old. Fifty-nine HCWs (30.26%) were 30–39 years old. Thirty-three HCWs (16.92%) were 40–49 years old. As for the BMI, 44 HCWs (51.76%) who had TB disease had a BMI of 18.5–22.9. Regarding work duration, 50 HCWs (58.82%) with TB disease had worked at KCMH for less than 5 years. The work duration of HCWs between 6–30 years who were diagnosed with TB disease ranged from 3.53 to 11.76%. Moreover, from the sub-group analysis, 30 HCWs with TB disease had worked at the hospital within 2 years. (Table 1).

**Table 1:** Age (2003–2020), BMI (2012–2020), and duration of work (2012–2020) of HCWs with TB disease at KCMH.

	Case		Case			Case
Age	N (%)	BMI (kg/m^2^)	N (%)		Work duration	N (%)
less than 20 years	4 (2.05)	less than 18.5	14 (16.47)		Less than 5 years	50 (58.82)
20–29 years	84 (43.08)	18.5–22.9	44 (51.76)		6–10 years	4 (4.71)
30–39 years	59 (30.26)	23–24.9	13 (15.29)		11–15 years	10 (11.76)
40–49 years	33 (16.92)	25–29.9	13 (15.29)		16–20 years	4 (4.71)
50–59 years	10 (5.13)	more than 30	1 (1.18)		21–25 years	8 (9.41)
more than 60 years	5 (2.56)				26–30 years	3 (3.53)
					more than 30 years	6 (7.06)
Total	195 (100.00)	Total	85 (100.00)		Total	85 (100.00)

Forty-four HCWs (51.76%) had drug sensitive TB (DS-TB). Twenty-one HCWs (24.70%) had drug resistant TB (DR-TB) of which 7 (33.33%) had multidrug resistant TB (MDR-TB). Twenty cases (23.53%) had negative result for TB via the culture. These cases were clinically diagnosed or had a positive PCR result. Most HCWs with TB disease were healthy (without comorbidity) at their annual health checkup but 13 (36.11%) of the healthy group had DR-TB. None of the HCW with TB disease had HIV infection. However, due to the small sample size, the relationship between underlying disease and DR-TB is not clear. Moreover, 83.33% of HCWs with TB disease who had previous history of TB disease had MDR-TB and pre-extensively drug resistant TB (Pre- XDR TB) (Table 2). All HCWs reported that they had been exposed to TB patients prior to their own diagnosis of TB disease. Baseline tuberculin skin test or IGRA is not routinely performed in all HCWs in high TB endemic country like Thailand, especially in early 2000, and did not collect their results during retrospective questionnaire. Thus, reports of biomarker in latent TB infection (LTBI) were missing in our study. According to the retrospective self-report and medical record review of the HCWs who had TB disease since 2013, none of the HCWs were investigated for LTBI nor treated for LTBI.

**Table 2:** Underlying diseases and drug resistant patterns of TB disease in HCWs at KCMH from 2012–2020.

Underlying disease	Drug resistance TB (N, (%))			
	No	Yes	N/A	total
No comorbidity	36 (54.55)	13 (19.70)	17 (25.76)	66
Comorbidity*	8 (42.11)	8 (42.11)	3 (15.79)	19
				85
DM	6 (46.15)	4 (30.77)	3 (15.09)	13
HT	3 (60.00)	1 (20.00)	1 (20.00)	5
DLP	3 (75.00)	0	1 (25.00)	4
Alcoholism	0	1 (100.00)	0	1
CKD	0	1 (100.00)	0	1
CVA	0	1 (100.00)	0	1
SLE	0	1 (100.00)	0	1
VHD	1 (100.00)	0	0	1
CA breast	1 (100.00)	0	0	1
HBV	1 (100.00)	0	0	1
Previous history of tuberculosis				
No	43 (54.33)	16 (20.25)	20 (25.32)	79
Yes	1 (16.67)	5 (83.33)	0	6

*HCW may have more than single comorbidity.

N/A: Not available DM: Diabetes, HT: Hypertension, DLP: Dyslipidemia, CKD: Chronic kidney disease, CVA: Cerebrovascular disease, SLE: Systemic lupus erythematous, VHD: Valvular heart disease, HBV: Chronic hepatitis B virus infection.

The Department of Medicine had the highest proportion of TB disease, up to 46 HCWs (23.59%). The supportive department (e.g., housekeepers, transporters, security guards) had 38 HCWs with TB disease (19.49%), followed by department of surgery (29 HCWs,14.87%) and medical students (16 students, 8.21%).

According to the incidence rate by specific profession, Radiological technician had the highest average incidence of TB disease at 429 per 100,000 radiological technicians with 95% confidential interval [CI], -107 to 964, (incidence was 0, 14, 0, 0, 0, 21, 0, 0 per 100,000 radiological technicians respectively), followed by supportive teams (e.g., housekeepers, transporters, security guards) (average 241 per 100,000 supportive teams with 95% CI, 71 to 412), scientists (e.g., laboratory technicians/scientists, researchers, blood bank teams) (average 205 per 100,000 scientists with 95% CI, -24 to 436), physicians (average 193 per 100,000 physicians with 95% CI, 28 to 359), assistant nurses (average 176 per 100,000 assistant nurses, with 95% CI, -17 to 368), nurses (average 157 per 100,000 nurses with 95% CI, -3 to 317), and pharmacists (average 95 per 100,000 pharmacists, with 95% CI, -143 to 334). The incidence of TB among specific profession was also calculated from 2013 to 2020 because there was insufficient information prior to 2013 (Table 3). The information used to calculate the incidence of TB among specific profession was the same as that used to calculate the overall TB incidence. It should be noted that the incidence of physical therapist could not be ascertained.

**Table 3:** Department of work and profession of HCWs with TB disease at KCMH from 2003–2020.

Department	Case	Occupation	Case	Incidence* (95% CI)
	N (%)		N (%)	
Medicine	46 (23.59)	Radiological tech.	8 (4.10)	429 (-107 to 964)
Support	38 (19.49)	Support	36 (18.46)	241 (71 to 412)
Surgery	29 (14.87)	Scientist	6 (3.07)	205 (-24 to 436)
Medical student	16 (8.21)	Physician	62 (31.79)	193 (28 to 359)
Radiology	12 (6.15)	Assistant nurse	25 (12.82)	176 (-17 to 368)
Emergency	11 (5.64)	Nurse	53 (27.18)	157 (-3 to 317)
Pedriatric	11 (5.64)	Pharmacist	2 (1.03)	95 (-143 to 334)
Laboratory	6 (3.08)	Physical therapist	2 (1.03)	0
OPD	6 (3.08)	Missing data	1 (0.51)	N/A
OBS-GYN	6 (3.08)			
Anesthesia	4 (2.05)			
Forensic	2 (1.03)			
Rehabilitation	2 (1.03)			
Pharmacist	2 (1.03)			
Orthopedic	1 (0.51)			
Nursing collage	1 (0.51)			
Missing data	2 (1.03)			
Total	195 (100.00)	Total	195 (100.00)	

* Incidence per 100,000 professions specific HCWs was calculated from 2013–2020.

Due to the large number of TB in physicians and nurses, the authors performed a sub-group analysis that was related to duration of work in years. At the first time, the category was divided every 5 years of working experience. Surprisingly, the proportion of the first 5 years was extremely high compared to the other time points. Therefore, the authors divided the first category into the first 2 years and third to fifth years to extract the difference between year of working in the early phase as extra columns. Eighteen physicians with TB disease (75%) had worked at KCMH within 2 years and 6 nurses with TB disease (23.07%) had worked at KCMH within 2 years. None of the physicians were diagnosed with TB disease since 2013. None of the physicians or nurses were diagnosed with TB after 35 years of work since 2013. In the subgroup analysis, the incidence of specific subgroup (e.g., residency program, fellowship program, student program, and nurses) was calculated by annual HCWs with TB disease and annual total HCWs in their specific program. According to the physician subgroup, the residency program had the highest TB incidence of 688 per 100,000 residents (95% CI, 273 to 1,102), followed by the fellowship program and medical students (665 per 100,000 physicians with 95% CI, 332 to 997 and 54 per 100,000 physicians with 95% CI, -122 to 230, respectively) (Table 4).

**Table 4:** Sub-group analysis of duration of work and profession of HCWs with TB disease at KCMH from 2012–2020.

Occupation		Work duration (years)								Total	Incidence* (95% CI)
		within 2	3–5	6–10	11–15	16–20	21–25	26–30	31- 35		
physicians	Residents	10	2	0	0	0	0	0	0	12	688 (273 to 1,102)
	Fellows	6	2	0	0	0	0	0	0	8	665 (332 to 997)
	Medical students	2	2	0	0	0	0	0	0	4	54 (-122 to 230)
	Total	18	6	0	0	0	0	0	0	24	193 (28 to 359)
Nurses		6	7	1	4	1	4	2	1	26	157 (-3 to 317)

* Incidence per 100,000 professions specific HCWs was calculated from 2013–2020.

According to the characteristic of TB disease among HCWs in 2012 to 2020, there was 85 HCWs with TB disease. One hundred and ten HCWs who were diagnosed with TB disease before 2012 had insufficient data to be classified to have the manifestation. According to the chest radiography, 62 HCWs with TB disease (83.78%) had lobar infiltration. Seven HCWs (9.46%) had cavitary lesion and six HCWs (8.11%) had pulmonary nodule. In additional, four HCWs (5.41%) had both lobar infiltration and pleural effusion but three (4.05%) had only pleural effusion. However, two HCWs (2.7%) with TB disease had normal chest radiography but were highly suspicious to have TB based on the clinical data; after further investigation, their sputum PCR was positive for TB. For microbiological evidence, sputum acid fast strain (AFB) was detected in only 22 of those diagnosed with TB disease (29.73%). From the sputum PCR (polymerase chain reaction) (61 HCWs; 82.43%) and sputum culture (63 HCWs; 85.14%), the detection for TB were higher than sputum AFB. Two HCWs were diagnosed by histopathology. Twenty of 85 HCWs (23.53%) were treated based on the clinical and radiological findings which were compatible with TB disease and responded to anti TB treatment. Thirty-six HCWs (42.3%) with TB disease had chronic cough for more than 2 weeks. Four HCWs (4.71%) had non-massive hemoptysis and three HCWs (3.53%) had pleuritis chest pain. Only one HCW (1.18%) had prolonged fever and weight loss. However, 37 HCWs (43.53%) were asymptomatic and detected to have TB disease when they had their annual chest x-rays. In the asymptomatic group, 3 of 37 HCWs (8.10%) had positive acid-fast bacilli, but 22 of 37 HCWs (59.45%) had positive PCR and 21 of 37 HCWs (56.76%) had positive sputum culture for TB. All HCWs who were diagnosed with TB disease were cured after they completed their treatment. No one was lost to follow-up or died during treatment.

## Discussion

This study is the second study conducted at KCMH, since the first study at KCMH was published in 2005; and is the fourth study conducted in Thailand [8–10]. The authors tried their best to gather all available data of TB from the HCWs from 2003–2020 (18 years worth of data). However, most of the analysis was done for the last 8 years (since 2013). Nevertheless, this cross-sectional study has the longest duration compared to other studies conducted in Thailand. This study assessed the incidence of TB in HCWs, demographic data, and characteristics of TB in HCWs. The incidence of overall TB at KCMH was 164 per 100,000 HCWs (95% CI, 107 to 220) which was slightly higher than the general Thai population (153 per 100,000 population with 95% CI, 116 to 195) in absolute numbers but not statistically significant. The incidence of MDR-TB was 15 per 100,000 HCWs (95% CI, 3 to 27) compared to 5.7 per 100,000 population (95% CI, 3.3 to 8.8) in the general Thai population [1]. The incidence from this study was different from the findings from 3 previous studies conducted in Thailand (KCMH, Ramathibodi, and Chiang Mai Hospital), Taiwan [11], South Africa [12] and worldwide systematic reviews [6,7], however, the incidence rate of tuberculosis in HCWs was significantly higher than the general population. In South Africa, 1 of the 14 countries to have high TB burden, the incidence of TB disease among HCWs was nearly double of that in their general population in 2010, (1,958 per 100,000 person- years compared to 981 per 100,000 person- years) [12]. In a later study, the incidence of TB in HCWs in South Africa was different according to local TB incidence [13]. On the other hand, the TB incidence of HCWs in the USA was lower than the general population [14].

At KCMH, the incidence of TB gradually increased since 2015 to a peak of 300 per 100,000 HCWs in 2019. There are 3 possible hypotheses to explain the high incidence. First, there is a high TB burden in the country and since KCMH is a referral center, therefore, there are more TB and MDR-TB patients compared to the general population. Second, due to the advanced technology such as molecular techniques, thus, this can increase the diagnostic yield compared to other tools used in the general hospital. Third, after the establishment of an active surveillance system by the infectious control committee, the detection of TB has increased. In 2016, the committee set up an air borne isolation–TB clinic that is a one-stop clinic and annual health checkup were imperative in identifying TB patients. This active case finding was implemented and found to be very effective. When TB was detected in an abnormal chest radiography, the person was called back for further investigation. HCWs with negative initial microbiological test were scheduled to induce sputum or have a bronchoscope specimen collection to increase the molecular diagnostic yield. The active surveillance resulted in early detection and treatment especially in asymptomatic HCWs who were able to spread TB to others unintentionally. On the other hand, the systems also raised the overall incidence of TB among HCWs in KCMH but would be beneficial in the long run. Therefore, active surveillances were a cornerstone of secondary prevention to find a new TB case, especially in asymptomatic HCWs. In order to decrease TB incidence among HCWs as primary prevention, education was provided which resulted in high awareness of TB and proper usage of personal protective equipment.

As a result of this, the incidence of TB in 2020 has decreased to 160 per 100,000 HCWs which is almost the same as in the general Thai population. This decrease may be due to many factors. For example, in the last 5 years, KCMH has established a strict TB control policy for both administrative control and environmental control. Second, active case finding in HCWs was implemented which increased the use of molecular diagnosis to early detect TB, resulting in preventing the spread of TB among HCWs. Last, it is also possible that the new normal lifestyles due to the COVID-19 pandemic, such as wearing a face mask and practicing social distancing, could have prevented the spread of TB among HCWs. However, it should be noted that this was seen only in 1 year. Therefore, additional study should be conducted post COVID-19 to see if the incidence of TB is still low or not because of the new normal practices. In addition, the study should have a longer follow-up period. The aim of this study is to lower the incidence of TB to less than 100 per 100,000 HCWs.

As mentioned above, the overall incidence of TB at KCMH was 164 per 100,000 HCWs with 95% confidential interval, [CI], 107 to 220. The 95% CI covered the incidence rate of the general population in Thailand, which was 153 per 100,000 population. Therefore, our study did not have enough power to declare that working at the hospital was a statistically significant risk factor for developing TB disease. The authors suggest that additional study should be conducted with a larger sample size or have a longer period of study which may be able to answer this question. However, KCMH is a university hospital and a referral center that is adequately supplied with personal protective equipment, N-95 respirator, and has an airborne insolation unit for HCWs and patients with TB compared to the general hospital in a resource limited setting. Further study should be conducted in a general hospital to assess the incidence rate and risk factors for developing TB among HCWs. It is possible that the incidence rate and risk factors in the general hospital may be different compared to our hospital. The incidence of TB was quite high in the past. However, in 2016, the committee set up an air borne isolation unit–TB clinic that is a one-stop clinic and provides annual health checkup. These things are imperative in identifying TB patients. This active case finding was implemented and found to be very effective. Therefore, the incidence decreased tremendously in the last 5 years, almost the same as in the normal population.

Aside from that, previous history of TB and diabetes mellitus (DM) were high in proportion to DR-TB. Other factors, such as alcoholism, chronic kidney disease (CKD), systemic lupus erythematosus (SLE), and cerebrovascular event, could not confirm its association to DR-TB due to small sample size. It is recommended that a case control study with a larger sample size should be done to prove this hypothesis and calculate the relative risks.

During the study, the participants had to complete a retrospective questionnaire. This questionnaire assessed the information pertaining to TB exposure from other TB patients up to the time the participants were diagnosed with TB disease. But baseline tuberculin skin test or IGRA was not routinely performed in all HCWs in high TB endemic country like Thailand. Molecular epidemiological analysis including DNA fingerprints of isolated TB from patients and HCWs also was not routinely performed in our study. Therefore, we cannot provide definitive evidence that HCWs acquired TB through nosocomial transmission from TB patients.

The Department of Medicine had the highest proportion of TB disease among HCWs. A newly diagnosed patient with TB disease in any department was transferred to an airborne isolation unit of the Department of Medicine. Moreover, most of the patients in the medicine ward had an immunosuppressive state so the TB was reactivated. Therefore, HCWs in the Department of Medicine were more likely to spread TB disease to the patients in the medicine ward who were susceptible to acquiring TB infection. The authors had limited access to the incidence of TB in that department because annual health checkup of the HCW was not done annually.

The second highest proportion of HCWs who were diagnosed with TB disease exhibited no symptoms. Therefore, annual health checkup is essential for early detection. PCR is a highly sensitive technique in detecting TB in Thailand [15], but its availability may be limited. Further study on sensitivity and specificity of PCR for TB in HCWs should be done in a larger sample size using various specimens such as spotted sputum, induced sputum, and bronchoalveolar lavage. Comparisons of the different types of specimens used should also be done to see which specimens have a higher PCR sensitivity rate.

For this study, there was a higher proportion of HCWs with TB disease were young and had a short duration of work at KCMH. These HCWs were frontline workers and had a higher chance to become infected with TB. Not only that, but these HCWs have little experience in managing patients with TB and have little knowledge on TB prevention, hence, resulting in higher prevalence of TB among these HCWs. Aside from this, other professional HCWs exposed to TB patients and infectious materials were radiological technicians and scientists. Other professional HCWs, including supportive teams, housekeepers, transporters, and office workers, were also exposed to TB patients without knowing the diagnosis due to patient confidentiality issue. Concordant with the previous systematic review in low to middle income countries, a higher risk of acquiring TB was associated with certain work locations (laboratory, internal medicine, and emergency facilities) and occupational categories (radiology technicians, patient attendants, nurses, ward attendants, paramedics, and clinical officers) [6]. Thus, education and awareness of TB among HCWs, administrative system, standard precaution, and active surveillance play an important role in reducing the incidence of TB in HCWs.

Due to the high numbers of TB disease in physicians and nurses as mentioned earlier, the authors performed subgroup analysis according to the profession of the HCWs. The residency program had the highest incidence of TB disease in the physician subgroup. Nurses often spend more time with patients than physicians which made them at greater risk of acquiring TB infection. In contrast, the incidence of TB disease in nurses was lower than the physicians, especially in the first 2 years which might be due to better awareness of TB. Additional study should be conducted and stratify the nurses according to their subspecialty as done with the physicians. It is unclear whether the TB disease was a recent infection or reactivation. For new TB infection, the HCWs were at the frontline and exposed to TB patients while managing them in the emergency unit. As for reactivation, it is possible that all residents had interned in the rural area for at least 3 years, and might have been infected with primary TB so when they became residents, their immune system may be suppressed due to high workload, lack of adequate sleep and stress which can reactivate TB. Quantitative TB immunologic study (e.g., IGRA) should be conducted in new physicians during the training program to prove this hypothesis. Future study should assess TB biomarkers that can distinguish between reactivation from latent infection and new infection among residents.

A major limitation of our study is that the data prior to 2013 were missing when the hospital changed their system from paper-based medical records to electronic medical records as previously mentioned. Nevertheless, this study analyzed all available data; therefore, the incidence and some variables have been calculated since 2013. The data available was one of the limitations of the study. This resulted in high standard deviation which caused the 95% confidential interval range to be wide in our study. We recommend that a longitudinal study with a larger sample size should be conducted.

In conclusion, the findings from this study will help the country to control the spread of TB among HCWs. Furthermore, the authors are committed to improve KCMH’s administrative system to reduce the incidence of TB so that it is lower than the general population.

## Supporting information

S1 Data (XLSX)

## References

[1] Global Tuberculosis report 2019: World Health Organization; [June 3, 2021]. Available from: https://www.who.int/teams/global-tuberculosis-programme/tb-reports/global-report-2019#:~:text=The%20Global%20TB%20Report%202019,in%20202%20countries%20and%20territories.

[2] Antonucci G, Girardi E, Raviglione MC, Ippolito G. Risk factors for tuberculosis in HIV-infected persons. A prospective cohort study. The Gruppo Italiano di Studio Tubercolosi e AIDS (GISTA). JAMA. 1995;274(2):143–8. Epub 1995/07/12. doi: 10.1001/jama.274.2.143 . 7596002

[3] Cowie RL. The epidemiology of tuberculosis in gold miners with silicosis. Am J Respir Crit Care Med. 1994;150(5 Pt 1):1460–2. Epub 1994/11/01. doi: 10.1164/ajrccm.150.5.7952577 . 7952577

[4] Rieder HL, Cauthen GM, Comstock GW, Snider DE Jr., Epidemiology of tuberculosis in the United States. Epidemiol Rev. 1989;11:79–98. Epub 1989/01/01. doi: 10.1093/oxfordjournals.epirev.a036046 . 2680563

[5] Narasimhan P, Wood J, Macintyre CR, Mathai D. Risk factors for tuberculosis. Pulm Med. 2013;2013:828939. doi: 10.1155/2013/828939 Epub 2013 Feb 12. ; PMCID: PMC3583136. 23476764 PMC3583136

[6] Joshi R, Reingold AL, Menzies D, Pai M. Tuberculosis among health-care workers in low- and middle-income countries: a systematic review. PLoS Med. 2006;3(12):e494. Epub 2006/12/30. doi: 10.1371/journal.pmed.0030494 ; PubMed Central PMCID: PMC1716189. 17194191 PMC1716189

[7] Baussano I, Nunn P, Williams B, Pivetta E, Bugiani M, Scano F. Tuberculosis among health care workers. Emerg Infect Dis. 2011;17(3):488–94. Epub 2011/03/12. doi: 10.3201/eid1703.100947 ; PubMed Central PMCID: PMC3298382. 21392441 PMC3298382

[8] Inchai J, Liwsrisakun C, Bumroongkit C, Euathrongchit J, Tajarernmuang P, Pothirat C. Tuberculosis among Healthcare Workers at Chiang Mai University Hospital, Thailand: Clinical and Microbiological Characteristics and Treatment Outcomes. Jpn J Infect Dis. 2018;71(3):214–9. Epub 2018/05/02. doi: 10.7883/yoken.JJID.2017.274 . 29709976

[9] Jiamjarasrangsi W, Hirunsuthikul N, Kamolratanakul P. Tuberculosis among health care workers at King Chulalongkorn Memorial Hospital, 1988–2002. Int J Tuberc Lung Dis. 2005;9(11):1253–8. Epub 2005/12/13. . 16333934

[10] Pongwittayapanu P, Anothaisintawee T, Malathum K, Wongrathanandha C. Incidence of Newly Diagnosed Tuberculosis among Healthcare Workers in a Teaching Hospital, Thailand. Ann Glob Health. 2018;84(3):342–7. Epub 2019/03/06. doi: 10.29024/aogh.2304 ; PubMed Central PMCID: PMC6748236. 30835396 PMC6748236

[11] Pan SC, Chen YC, Wang JY, Sheng WH, Lin HH, Fang CT, et al. Tuberculosis in Healthcare Workers: A Matched Cohort Study in Taiwan. PLoS One. 2015;10(12):e0145047. Epub 2015/12/19. doi: 10.1371/journal.pone.0145047 ; PubMed Central PMCID: PMC4683009. 26679188 PMC4683009

[12] Tudor C., Van der Walt M., Margot B. et al. Tuberculosis among health care workers in KwaZulu-Natal, South Africa: a retrospective cohort analysis. BMC Public Health 14, 891 (2014). doi: 10.1186/1471-2458-14-891 25174848 PMC4161912

[13] Grobler L, Mehtar S, Dheda K, Adams S, Babatunde S, van der Walt M, et al. The epidemiology of tuberculosis in health care workers in South Africa: a systematic review. BMC Health Serv Res. 2016;16(1):416. Epub 2016/08/22. doi: 10.1186/s12913-016-1601-5 ; PubMed Central PMCID: PMC4992336. 27544429 PMC4992336

[14] Mongkolrattanothai T, Lambert LA, Winston CA. Tuberculosis among healthcare personnel, United States, 2010–2016. Infect Control Hosp Epidemiol. 2019;40(6):701–4. Epub 2019/04/24. doi: 10.1017/ice.2019.76 ; PubMed Central PMCID: PMC6611159. 31012401 PMC6611159

[15] National Tuberculosis control Program Guideline, Thailand 2018: Pediatric Infectious Disease Society of Thailand; [June 3, 2021]. Available from: https://www.pidst.or.th/A641.html?action=download&file=750_NationTBguideline2018.pdf.

